# On the Time to Buffer Overflow in a Queueing Model with a General Independent Input Stream and Power-Saving Mechanism Based on Working Vacations

**DOI:** 10.3390/s21165507

**Published:** 2021-08-16

**Authors:** Martyna Kobielnik, Wojciech Kempa

**Affiliations:** Department of Mathematics Applications and Methods for Artificial Intelligence, Faculty of Applied Mathematics, Silesian University of Technology, ul. Kaszubska 23, 44-100 Gliwice, Poland; wojciech.kempa@polsl.pl

**Keywords:** energy saving, queueing model, time to buffer overflow, transient analysis, working vacation policy

## Abstract

A single server GI/M/1 queue with a limited buffer and an energy-saving mechanism based on a single working vacation policy is analyzed. The general independent input stream and exponential service times are considered. When the queue is empty after a service completion epoch, the server lowers the service speed for a random amount of time following an exponential distribution. Packets that arrive while the buffer is saturated are rejected. The analysis is focused on the duration of the time period with no packet losses. A system of equations for the transient time to the first buffer overflow cumulative distribution functions conditioned by the initial state and working mode of the service unit is stated using the idea of an embedded Markov chain and the continuous version of the law of total probability. The explicit representation for the Laplace transform of considered characteristics is found using a linear algebra-based approach. The results are illustrated using numerical examples, and the impact of the key parameters of the model is investigated.

## 1. Introduction

Evidently, the problem of reducing energy consumption is global. This results in large-scale research on algorithms supporting power-saving control and the accompanying technical solutions. Energy-saving solutions are particularly desired in the area of computer and telecommunications networks, which is related to the rapidly growing share of wireless transmissions. Wireless network components, such as sensor network sensors, are powered by batteries. Such networks are designed to constantly monitor the air temperature, humidity, road traffic intensity, etc. According to their purpose, e.g., to warn about fire hazards, sensors (network nodes) are often located in hard-to-reach places.

Limiting energy consumption and, consequently, extending the possibility of powering a node from a single battery is, therefore, of key importance in ensuring reliable data transmission and the associated security. Queueing theory is widely used in traffic modelling in energy-efficient packet networks. Indeed, queueing models, especially those with a finite capacity of accumulating buffer in which a mechanism limiting the operation of the service station has been implemented (in the case of, e.g., low-intensity traffic), can be effectively used in the process of controlling the QoS (Quality of Service) and the energy consumption of individual nodes. The knowledge of stochastic characteristics changing in time, such as the distribution of the queue length, queueing delay, or the time to buffer overflow, allows for ongoing monitoring of the system and, thus, for control of the transmission quality.

The concept of a queue model was proposed for the first time in [1], in which a service station remains unavailable for job service for some time. Queueing systems with server vacations quickly gained popularity, and many new models and a whole range of analytical results concerning such models appeared in the literature. An exhaustive study on queueing models with different types of vacation policies can be found, e.g., in [2] or in survey papers [3,4].

In [5], a model was proposed in which—instead of temporarily suspending the service—the server processes jobs with different intensities (speeds) depending, e.g., on the intensity of incoming traffic. On the one hand, such a policy, called a working vacation (WV), allows for energy savings (caused by a temporary reduction in job service intensity), and on the other hand, allows for better control of the queue length and reduces the risk of serial job loss. Moreover, it allows to redirect the unused resources for other tasks, e.g., for maintenance purpose or for redirecting traffic from other nodes.

Under a single working vacation policy, the service station takes only one working vacation when the queue is emptied. An alternative is multiple vacation policy, in which successive single working vacations are initialized as far as at least one job waiting in the queue is detected. In [6], a model of working vacation was studied in the context of energy saving and latency control in wireless sensor networks. The authors introduced a two-threshold working vacation policy, which is a combination of a vacation and working vacation policy.

In [7], the two–channel model M/M/2 with WV, negative customers, feedback, and N-strategy was proposed to reduce the energy consumption in wireless communication networks. The energy saving capabilities of WV models were also discussed from the cloud platform point of view, see, e.g., [8,9].

An M/G/1-type queueing model with single server working vacations was studied in [10], where the stationary queue-size distribution was obtained via the supplementary variable technique and matrix-analytic approach. A G/M/1 infinite-buffer system with a single working vacation policy was investigated in [11,12]. A discrete-time model with single working vacations was analysed in [13], where interarrival times and service times are geometrically distributed.

In [14], a model with a general independent input stream and single working vacation policy is studied in the case of memoryless service time distribution (both exponential and geometrical). A M/G/1-type queueing model with single working vacations and vacation interruption under a Bernoulli schedule was considered in [15].

In such a model, if there are jobs present in the system at the working vacation completion moment, the server can initialize the next working vacation period (with probability *p*) or it can return to the normal mode (with probability 1−p). The joint distribution of the steady-state queue size and service status is then derived by using the supplementary variable technique.

A similar model was investigated in [16], where the sojourn time distribution is obtained. Recently, in [17], the steady-state characteristics of a Markovian queue with working vacations and breakdowns were studied using the spectral expansion method. In [18], a discrete-time model with general batch input and geometric service time with multiple working vacations was studied with the supplementary variable technique.

A model with a single working vacation, customer impatience, and catastrophes was analysed in [19]. The steady-state distribution of the system size for a model with customer impatience and server breakdowns was obtained in [20]. Additionally, the authors solved a profit optimization problem using a particle swarm optimization algorithm.

As can be seen, the vast majority of the results obtained for models with working vacations concern the steady state of the system. In practice, however, there are often situations in which stationary analysis is insufficient. This is the case, for example, when observing a system immediately after its opening (when its steady state has not yet been established), after changing the control mechanism, or after removing a failure.

In the case of low traffic intensity (which is typical for, e.g., sensor networks), system stabilization may take a long time and, consequently, the steady state ceases to be an indicator of system operation. There are few results for the transient state, especially for models with a general input stream. In [21], the study of a model with general input and phase type service was carried out using a simulation-based approach.

In [22], the transient results for the number served during a busy period in a GI/M/1/N queue was obtained by approximating the interarrival distribution according to a two-phase Cox distribution. A more general model, G/G/m/m+K, was studied in [23] using the diffusion approximation technique.

With regards to working vacation policy, most of the papers concern only a M/M/1 queue, e.g., [24,25]. A M/M/1 queue with working vacation and impatient customers was studied in [25]. The transient system size probabilities were obtained using a continued fractions approach. In [24], the transient solution was found by solving differential equations using the Runge–Kutta algorithm.

In [26], the transient behaviour of a finite-capacity model with a general independent input flow of jobs and single working vacation policy was investigated. Using an analytic approach based on the idea of an embedded Markov chain and linear algebra, the compact-form representation for the Laplace transform of the queue-size distribution conditioned by the initial buffer state was derived.

An energy-saving mechanism based on a threshold-controlled multiple vacation policy was considered in [27] as a model for the operation a wireless sensor network node. The Laplace transform representations were obtained for queue-size distribution at an arbitrary fixed time and for idle and processing periods.

Moreover, the compact-form formulae for the distributions of the idle and processing period duration were found. A mathematical model for the node of a wireless sensor network with discrete-time parameters was proposed in [28]. An explicit formula for the tail cumulative distribution function of the first buffer overflow period duration was obtained. Hence, the corresponding result for the next buffer overflow periods was found.

In this paper, we study a finite-buffer GI/M/1/N queueing model with general-type independent input flow of jobs, exponentially distributed service times, and a single working vacation policy. Applying an analytic approach based on the idea of embedded Markov chain and linear algebra, we find the closed-form representation for the Laplace transform of the time to the first buffer overflow distribution, conditioned by the initial system state and working mode of the service unit. The theoretical results are illustrated using numerical examples.

The remainder of the article is organized as follows. In Section 2, we provide a precise mathematical description of the considered queueing model and present an auxiliary algebraic result. In Section 3, systems of integral equations for conditional distributions of the time to the first buffer overflow, based on the idea of embedded Markov chain and the continuous version of the formula of total probability, are established for both the system start and operation in normal and working vacation mode. The closed-form solutions for corresponding systems written for Laplace transforms are found in Section 4. Section 5 is devoted to detailed numerical examples illustrating theoretical results. Finally, Section 6 contains a short conclusion.

## 2. Model Description and Auxiliary Result

Let us consider a GI/M/1/N/WV model, where the times between successive arrivals are independent random variables with a common cumulative distribution function (CDF) F(t), and the service times are exponential random variables with parameters μ and $\mu_{v}$ in normal mode and during a working vacation, respectively. The system is characterized by a finite buffer. At any given time, only *N* jobs can be present, namely one in the service unit and N−1 in the queue.

Every time the server finds the queue empty after the service completion epoch, it enters a single working vacation period, changing the service intensity to a lower value $\mu_{v}$, and stays in this mode for a period of time that is exponentially distributed with parameter α. When the WV period ends, the server returns to normal mode and normal service speed $\mu >\mu_{v}$.

Let X(t) be the number of jobs present in the system at a time epoch *t*. The random variable

$$\gamma =\min\left\{t\geq 0:X(t)=N\right\}$$

denotes the time to the first buffer overflow. Our goal is to determine the conditional CDFs of γ given the initial state and working mode of the server, i.e.,

$$T_{n}\left(t\right)=P\left\{\gamma \leq t|X(0)=n,Y(0)=0\right\},$$

and

$$T_{n}^{v}\left(t\right)=P\left\{\gamma \leq t|X(0)=n,Y(0)=1\right\},$$

where Y(t)=0 if at the time instant *t*, the server is in normal mode, and Y(t)=1, otherwise.

In the following subsections, a system of integral equations for $T_{n}\left(t\right)$ and $T_{n}^{v}\left(t\right),$n=0,1,…,N is stated using the formula of total probability and the method of embedded Markov chain. In the next section, the corresponding system for Laplace transforms of $T_{n}\left(t\right)$ and $T_{n}^{v}\left(t\right)$ is solved applying the method of potential.

For the rest of this paper, we use the following notations:

$$\begin{array}{cc} p_{i}\left(a\right) & =\frac{a^{i}}{i!}e^{-a},i=0,1,\ldots ,\\ E_{i,a}\left(t\right) & =1-\sum_{j=0}^{i-1}\frac{{\left(at\right)}^{i}}{i!}e^{-at},i=1,2,\ldots , \end{array}$$

i.e., $p_{i}\left(a\right)$ is the probability function of a Poisson distribution with parameter *a*, and $E_{i,a}\left(t\right)$ is the CDF of the Erlang distribution with a shape parameter *i* and a scale parameter *a*. We also assume that N>2,μ≠α, and $\alpha +\mu_{v}\neq \mu .$ If any of these assumptions are not satisfied, the model is simplified, and those cases are not taken into consideration.

The concept of potential random walk is introduced in [29] as a tool for the analysis of compound Poisson processes. In particular, it is proven in [29] (see also [30]) that each solution of the infinite-size system of linear equations of the form

(1) $$\begin{array}{cc} & \sum_{k=-1}^{n-2}a_{k+1}x_{n-k}-x_{n}=\theta_{n}, \end{array}$$

where n≥2, can be written as

(2) $$\begin{array}{cc} & x_{n}=MR_{n-1}+\sum_{k=2}^{n}R_{n-k}\phi_{k},\;n\geq 2, \end{array}$$

where M∈R, and the sequence $\left(R_{k}\right)$ is defined as indicated below.

Consider the following generating functions:

(3) $$\begin{array}{cc} & r\left(\theta \right)\overset{def}{=}\sum_{k=0}^{\infty }\theta^{k}R_{k} \end{array}$$

and

(4) $$\begin{array}{cc} & a\left(\theta \right)\overset{def}{=}\sum_{k=0}^{\infty }\theta^{k}a_{k}, \end{array}$$

where |θ|<1.

It can be shown ([29,30]) that the following representation is true:

(5) $$\begin{array}{cc} & r\left(\theta \right)=\frac{\theta }{a(\theta )-\theta }. \end{array}$$

As a consequence, applying Maclaurin’s expansion, we obtain

(6) $$\begin{array}{cc} & R_{k}=\frac{1}{k!}\frac{d^{k}}{d\theta^{k}}\left(\frac{\theta }{a(\theta )-\theta }\right){|}_{\theta =0},\;k\geq 1. \end{array}$$

Equivalently (see [29,30]), the sequence $\left(R_{k}\right)$ can be defined in a recursive way as follows:

(7) $$\begin{array}{cc} & R_{0}=0,\;R_{1}={\left(a_{0}\right)}^{-1},\\ & R_{k}=R_{1}\left(R_{k-1}-\sum_{j=0}^{k-1}a_{j+1}R_{k-1-i}\right), \end{array}$$

where k≥2.

## 3. Transient Equations for the Time to the Buffer Overflow Distribution

### 3.1. Server Starting in Normal Mode

When the buffer is empty and in normal mode upon opening, we have

(8) $$\begin{array}{cc} T_{0}\left(t\right) & =\int_{0}^{t}T_{1}\left(t-x\right)dF\left(x\right). \end{array}$$

If the first arrival occurs before *t* (at some epoch *x*), then the probability that the buffer overflows before *t* can be expressed using the probability of the buffer overflow before t−x, given that there is one job present at the beginning, since the system renews at *x* and behaves like it has just started with one job present. If no new jobs enter the system before *t*, then there will clearly be no buffer overflow up to *t*.

For 1≤n<N, the following is true:

(9) $$\begin{array}{c} T_{n}\left(t\right)=S_{1}\left(t,n\right)+S_{2}\left(t,n\right). \end{array}$$

The summand $S_{1}\left(t,n\right)$ stands for the case of a new arrival before epoch *t*, and 0≤i<n jobs are finished before this event. Therefore, the system renews with n−i+1 jobs present. This summand can be expressed by

(10) $$\begin{array}{c} S_{1}\left(t,n\right)=\int_{0}^{t}\sum_{i=0}^{n-1}p_{i}\left(\mu x\right)T_{n-i+1}\left(t-x\right)dF\left(x\right). \end{array}$$

The second summand, $S_{2}\left(t,n\right),$ results from the case where all jobs are finished before the new one arrives. This means that the server must change its operation mode to WV. Then, with the probability $1-e^{-\alpha (x-y)}$, the system will switch back to the normal mode before the first arrival. Otherwise, the system will renew in WV mode:

(11) $$\begin{array}{c} S_{2}\left(t,n\right)=\int_{0}^{t}dF\left(x\right)\int_{0}^{x}\left[\left(1-e^{-\alpha (x-y)}\right)T_{1}\left(t-x\right)+e^{-\alpha (x-y)}T_{1}^{v}\left(t-x\right)\right]dE_{n,\mu }\left(y\right). \end{array}$$

The last considered case is n=N. It is clear that if *N* customers are present at the beginning, then the buffer overflow before any time instant *t* is a certain event; therefore,

(12) $$\begin{array}{c} T_{N}\left(t\right)=1. \end{array}$$

### 3.2. Server Starting in WV Mode

If the server starts in WV mode with no jobs, we have

(13) $$\begin{array}{cc} T_{0}^{v}\left(t\right) & =\int_{0}^{t}\left[\left(1-e^{-\alpha x}\right)T_{1}\left(t-x\right)+e^{-\alpha x}T_{1}^{v}\left(t-x\right)\right]dF\left(x\right). \end{array}$$

For 1≤n<N, the CDF $T_{n}^{v}\left(t\right)$ satisfies

(14) $$\begin{array}{c} T_{n}^{v}\left(t\right)=\sum_{i=1}^{5}S_{i}^{v}\left(t,n\right). \end{array}$$

In the first two summands, the case of the system still being in WV mode when the new job enters is considered. Allowing for the fact that not all of the customers were served before the first arrival, we have

(15) $$\begin{array}{c} S_{1}^{v}\left(t,n\right)=\int_{0}^{t}e^{-\alpha x}\sum_{i=0}^{n-1}p_{i}\left(\mu_{v}x\right)T_{n-i+1}^{v}\left(t-x\right)dF\left(x\right), \end{array}$$

and, given all of the jobs were finished before this arrival, we conclude that

(16) $$\begin{array}{c} S_{2}^{v}\left(t,n\right)=\int_{0}^{t}e^{-\alpha x}E_{n,\mu_{v}}\left(x\right)T_{1}^{v}\left(t-x\right)dF\left(x\right). \end{array}$$

For the remaining summands, we assume that the WV period ends before the first arrival epoch. Hence, we need to take into account the number of customers served in WV (*i*) and in normal mode (*j*).

If i+j<n, the following expression is obtained:

(17) $$\begin{array}{c} S_{3}^{v}\left(t,n\right)=\int_{0}^{t}dF\left(x\right)\int_{0}^{x}\alpha e^{-\alpha y}\sum_{i=0}^{n-1}p_{i}\left(\mu_{v}y\right)\sum_{j=0}^{n-i-1}p_{j}\left(\mu (x-y)\right)T_{n-i-j+1}\left(t-x\right)dy. \end{array}$$

When i=n at the arrival epoch, the system is operating in normal mode, since the system empties not in normal but in the WV period, and thus no new WV period is initialized; therefore,

(18) $$\begin{array}{c} S_{4}^{v}\left(t,n\right)=\int_{0}^{t}dF\left(x\right)\int_{0}^{x}\alpha e^{-\alpha y}E_{n,\mu_{v}}\left(y\right)T_{1}\left(t-x\right)dy. \end{array}$$

If the last job is finished after switching to normal mode, the system starts a new WV period, we need to consider both cases of normal and WV mode at the arrival instant, and we can write

(19) $$\begin{array}{cc} S_{5}^{v}\left(t,n\right)=\; & \int_{0}^{t}dF\left(x\right)\int_{0}^{x}\alpha e^{-\alpha y}\sum_{i=0}^{n-1}p_{i}\left(\mu_{v}y\right)\\ \cdot & \int_{0}^{x-y}\left[\left(1-e^{-\alpha (x-(y+u))}\right)T_{1}\left(t-x\right)+e^{-\alpha (x-(y+u))}T_{1}^{v}\left(t-x\right)\right]dE_{n-i,\mu }\left(u\right)dy. \end{array}$$

For n=N, we have

(20) $$\begin{array}{c} T_{N}^{v}\left(t\right)=1. \end{array}$$

## 4. Solution of the System of Equations for $T_{n}\left(T\right)$ and $T_{n}^{V}\left(T\right)$

In this section, the system (8)–(20) is solved. It is divided into two subsections depending on the state of the server at the beginning. First, the solutions of (8) and (9) are found. Next, the solutions of (13) and (14) are explicitly obtained and introduced to the former, which results in an explicit solution for the Laplace transforms of $T_{i}\left(t\right)$ and $T_{i}^{v}\left(t\right),\;i=0,\ldots ,N-1.$

### 4.1. Solution for the Normal Mode

Let us denote

$${\tilde{T}}_{n}\left(s\right)=\int_{0}^{\infty }e^{-st}T_{n}\left(t\right)dt,\;\;\tilde{F}\left(s\right)=\int_{0}^{\infty }e^{-st}dF\left(t\right).$$

Additionally, we introduce the following notation:

$$a_{i}\left(s\right)=\int_{0}^{\infty }e^{-st}p_{i}\left(\mu t\right)dF\left(t\right),\;\;b_{i}\left(s\right)=\int_{0}^{\infty }e^{-st}E_{i,\mu }\left(t\right)dF\left(t\right),$$

$$c_{i}\left(s\right)={\left(\frac{\mu }{\mu -\alpha }\right)}^{i}\int_{0}^{\infty }e^{-t(s+\alpha )}E_{i,\mu -\alpha }\left(t\right)dF\left(t\right).$$

Now, the Laplace transform of the system (8)–(12) can be written in the following form:

(21) $$\begin{array}{cc} {\tilde{T}}_{0}\left(s\right) & ={\tilde{T}}_{1}\left(s\right)\tilde{F}\left(s\right)\\ {\tilde{T}}_{n}\left(s\right) & =\sum_{i=0}^{n-1}{\tilde{T}}_{n-i+1}\left(s\right)a_{i}\left(s\right)+{\tilde{T}}_{1}\left(s\right)\left(b_{n}\left(s\right)-c_{n}\left(s\right)\right)+{\tilde{T}}_{1}^{v}\left(s\right)c_{n}\left(s\right),\; \end{array}$$

(22) $$\begin{array}{c} n=1,\ldots N-1 \end{array}$$

(23) $$\begin{array}{cc} {\tilde{T}}_{N}\left(s\right) & =\frac{1}{s} \end{array}$$

If we denote

(24) $$\begin{array}{c} \phi_{n}\left(s\right)={\tilde{T}}_{1}\left(s\right)\left(c_{n}\left(s\right)-b_{n}\left(s\right)\right)-{\tilde{T}}_{1}^{v}\left(s\right)c_{n}\left(s\right), \end{array}$$

we can rewrite system (22) for n=1,…,N−1 in the form

(25) $$\begin{array}{c} \sum_{i=-1}^{n-2}a_{i+1}\left(s\right){\tilde{T}}_{n-i}\left(s\right)-{\tilde{T}}_{n}\left(s\right)=\phi_{n}\left(s\right). \end{array}$$

We can observe that the former system has the same form as system (1), and thus the solution can be obtained using (2), which leads to the following representation:

(26) $$\begin{array}{c} {\tilde{T}}_{n}\left(s\right)=M\left(s\right)R_{n-1}\left(s\right)+\sum_{i=2}^{n}R_{n-i}\left(s\right)\phi_{i}\left(s\right),\;n\geq 2, \end{array}$$

where $R_{k}\left(s\right)$ is a sequence defined as follows (see (7)):

$$R_{0}\left(s\right)=0,\;\;R_{1}\left(s\right)=a_{0}^{-1}\left(s\right),\;\;R_{k+1}\left(s\right)=R_{1}\left(s\right)\left(R_{k}\left(s\right)-\sum_{i=0}^{k}a_{i+1}\left(s\right)R_{k-i}\left(s\right)\right),$$

and M(s) is some unknown function.

Taking n=1 in (22) and n=2 in (26), we can derive M(s) as

(27) $$\begin{array}{c} M\left(s\right)={\tilde{T}}_{1}\left(s\right)\left(1-b_{1}\left(s\right)+c_{1}\left(s\right)\right)-{\tilde{T}}_{1}^{v}\left(s\right)c_{1}\left(s\right). \end{array}$$

Now, the solution (26) can be rewritten in the form:

(28) $$\begin{array}{c} \begin{array}{cc} {\tilde{T}}_{n}\left(s\right) & ={\tilde{T}}_{1}\left(s\right)\left(R_{n-1}\left(s\right)+\sum_{i=1}^{n}R_{n-i}\left(s\right)\left(c_{i}\left(s\right)-b_{i}\left(s\right)\right)\right)\\ & -{\tilde{T}}_{1}^{v}\left(s\right)\sum_{i=1}^{n}R_{n-i}\left(s\right)c_{i}\left(s\right),\;n\geq 2. \end{array} \end{array}$$

Combining (22) and (28) for n=N−1 yields

(29) $$\begin{array}{cc} {\tilde{T}}_{1}\left(s\right)= & \frac{K(s)}{L(s)}{\tilde{T}}_{1}^{v}\left(s\right)+\frac{a_{0}\left(s\right)}{sL(s)}, \end{array}$$

where

(30) $$\begin{array}{cc} K(s)= & \sum_{i=1}^{N-2}\left(R_{N-i-1}\left(s\right)c_{i}\left(s\right)-a_{i}\left(s\right)\sum_{j=1}^{N-i}R_{N-i-j}\left(s\right)c_{j}\left(s\right)\right)+c_{N-1}\left(s\right),\\ L(s)= & \sum_{i=1}^{N-2}\left(R_{N-i-1}\left(s\right)\left(c_{i}\left(s\right)-a_{i}\left(s\right)-b_{i}\left(s\right)\right)-\sum_{j=1}^{N-i}R_{N-i-j}\left(s\right)\left(c_{j}\left(s\right)-b_{j}\left(s\right)\right)\right) \end{array}$$

(31) $$\begin{array}{cc} \; & +R_{N-2}\left(s\right)+c_{N-1}\left(s\right)-b_{N-1}\left(s\right). \end{array}$$

Using the former expression in (28), we can state the solution (depending on ${\tilde{T}}_{1}^{v}\left(s\right)$) in the form

(32) $$\begin{array}{c} \begin{array}{cc} {\tilde{T}}_{n}\left(s\right)= & {\tilde{T}}_{1}^{v}\left(s\right)\left(\frac{K(s)}{L(s)}R_{n-1}\left(s\right)+\sum_{i=1}^{n}R_{n-i}\left(s\right)\left(\frac{K(s)}{L(s)}\left(c_{i}\left(s\right)-b_{i}\left(s\right)\right)-c_{i}\left(s\right)\right)\right)\\ \; & +\frac{a_{0}\left(s\right)}{sL(s)}\left(R_{n-1}\left(s\right)+\sum_{i=1}^{n}R_{n-i}\left(c_{i}\left(s\right)-b_{i}\left(s\right)\right)\right). \end{array} \end{array}$$

### 4.2. Solution for the WV Mode

In this subsection, the solution for the case of the server starting in WV mode is obtained.

The Laplace transform of the system (13)–(20) is given by

(33) $$\begin{array}{cc} {\tilde{T}}_{0}^{v}\left(s\right)= & {\tilde{T}}_{1}\left(s\right)\left(\tilde{F}\left(s\right)-\tilde{F}\left(s+\alpha \right)\right)+{\tilde{T}}_{1}^{v}\left(s\right)\tilde{F}\left(s+\alpha \right),\\ {\tilde{T}}_{n}^{v}\left(s\right)= & \sum_{i=0}^{n-1}{\tilde{T}}_{n-i+1}^{v}A_{i}\left(s\right)+{\tilde{T}}_{1}^{v}\left(s\right)B_{n}\left(s\right) \end{array}$$

(34) $$\begin{array}{cc} + & \sum_{i=0}^{n-1}\sum_{j=0}^{n-i-1}{\tilde{T}}_{n-i-j+1}\left(s\right)C_{ij}\left(s\right)+{\tilde{T}}_{1}\left(s\right)D_{n}\left(s\right),\;n=1,\ldots ,N-1, \end{array}$$

(35) $$\begin{array}{cc} {\tilde{T}}_{N}^{v}\left(s\right)= & \frac{1}{s}, \end{array}$$

where

(36) $$\begin{array}{cc} A_{i}\left(s\right)= & \int_{0}^{\infty }e^{-t(s+\alpha )}p_{i}\left(\mu_{v}t\right)dF\left(t\right),\\ B_{i}\left(s\right)= & \int_{0}^{\infty }e^{-t(s+\alpha )}E_{i,\mu_{v}}\left(t\right)dF\left(t\right) \end{array}$$

(37) $$\begin{array}{cc} \; & +\alpha \sum_{j=0}^{i-1}{\left(\frac{\mu }{\mu -\alpha }\right)}^{i-j}\int_{0}^{\infty }e^{-t(s+\alpha )}\int_{0}^{t}p_{j}\left(\mu_{v}y\right)E_{i-j,\mu -\alpha }\left(t-y\right)dy\;dF\left(t\right), \end{array}$$

(38) $$\begin{array}{cc} C_{ij}\left(s\right)= & \alpha \int_{0}^{\infty }e^{-st}\int_{0}^{t}e^{-\alpha y}p_{i}\left(\mu_{v}y\right)p_{j}\left(\mu (t-y)\right)dydF\left(t\right),\\ D_{i}\left(s\right)= & \alpha \int_{0}^{\infty }e^{-st}\int_{0}^{t}e^{-\alpha y}E_{i,\mu_{v}}\left(y\right)dy\;dF\left(t\right)+\alpha \int_{0}^{\infty }e^{-st}\int_{0}^{t}p_{j}\left(\mu_{v}y\right) \end{array}$$

(39) $$\begin{array}{cc} \; & \cdot \sum_{j=0}^{i-1}\left(e^{-\alpha y}E_{i-j,\mu }\left(t-y\right)-{\left(\frac{\mu }{\mu -\alpha }\right)}^{i-j}E_{i-j,\mu -\alpha }\left(t-y\right)\right)dy\;dF\left(t\right). \end{array}$$

The solutions (29) and (32) can be introduced into (34), which leads to the following form:

(40) $$\begin{array}{cc} {\tilde{T}}_{n}^{v}\left(s\right) & =\sum_{i=0}^{n-1}{\tilde{T}}_{n-i+1}^{v}\left(s\right)A_{i}\left(s\right)+{\tilde{T}}_{1}^{v}\left(s\right)G_{n}\left(s\right)+H_{n}\left(s\right), \end{array}$$

(41) $$\begin{array}{cc} {\tilde{T}}_{N}^{v}\left(s\right) & =\frac{1}{s}, \end{array}$$

where (we set l=n−i−j for readability):

(42) $$\begin{array}{cc} G_{n}\left(s\right) & =B_{n}\left(s\right)+\frac{K(s)}{L(s)}D_{n}\left(s\right)+\sum_{i=0}^{n-1}\sum_{j=0}^{n-i-1}C_{ij}\left(s\right)\\ \cdot & \left(\frac{K(s)}{L(s)}R_{l}\left(s\right)+\sum_{k=1}^{l}R_{l-k+1}\left(s\right)\left(\frac{K(s)}{L(s)}\left(c_{k}\left(s\right)-b_{k}\left(s\right)\right)-c_{k}\left(s\right)\right)\right)\\ - & I\left\{n=N-1\right\}C_{00}\left(s\right)\left(\frac{K(s)}{L(s)}R_{N-1}\left(s\right)+\sum_{k=1}^{N-1}R_{N-k}\left(s\right)\left(\frac{K(s)}{L(s)}\left(c_{k}\left(s\right)-b_{k}\left(s\right)\right)-c_{k}\left(s\right)\right)\right), \end{array}$$

(43) $$\begin{array}{cc} H_{n}\left(s\right) & =\frac{a_{0}\left(s\right)}{sL(s)}\\ \cdot & \left(D_{n}\left(s\right)+\sum_{i=0}^{n-1}\sum_{j=0}^{n-i-1}C_{ij}\left(s\right)\left(R_{l}\left(s\right)+\sum_{k=1}^{l+1}R_{l-k+1}\left(s\right)\left(c_{k}\left(s\right)-b_{k}\left(s\right)\right)\right)\right)\\ - & I\left\{n=N-1\right\}C_{00}\left(s\right)\left(R_{N-1}\left(s\right)+\sum_{k=1}^{N-1}R_{N-k}\left(s\right)\left(c_{k}\left(s\right)-b_{k}\left(s\right)\right)\right), \end{array}$$

for n<N−1, where $I\left\{\cdot \right\}$ is an indicator function. The system (40) can be solved with the approach used in previous subsection. It can be rewritten in the following form (compare (1)):

(44) $$\begin{array}{c} \sum_{i=-1}^{n-2}A_{i+1}\left(s\right){\tilde{T}}_{n-i}^{v}\left(s\right)-{\tilde{T}}_{n}^{v}\left(s\right)=\Phi_{n}\left(s\right),\;\;n=1,2,\ldots ,N-1, \end{array}$$

where $\Phi_{n}\left(s\right)=-{\tilde{T}}_{1}^{v}\left(s\right)G_{n}\left(s\right)-H_{n}\left(s\right).$

Now, the solution is found in the form (see (2)):

(45) $$\begin{array}{cc} {\tilde{T}}_{n}^{v}\left(s\right) & =M^{v}\left(s\right)R_{n-1}^{v}\left(s\right)+\sum_{i=2}^{n}R_{n-i}^{v}\left(s\right)\Phi_{i}\left(s\right),\;\;n\geq 2, \end{array}$$

where $M^{v}\left(s\right)$ is some function, and $R_{n}^{v}\left(s\right)$ is a sequence:

$$R_{0}^{v}\left(s\right)=0,\;\;R_{1}^{v}\left(s\right)=A_{0}^{-1}\left(s\right),\;\;R_{k+1}^{v}\left(s\right)=R_{1}^{v}\left(s\right)\left(R_{k}^{v}\left(s\right)-\sum_{i=0}^{k}A_{i+1}\left(s\right)R_{k-i}^{v}\left(s\right)\right).$$

Comparing (40) for n=1 with (45) for n=2, we find

(46) $$\begin{array}{c} M^{v}\left(s\right)={\tilde{T}}_{1}^{v}\left(s\right)\left(1-G_{1}\left(s\right)\right)-H_{1}\left(s\right). \end{array}$$

Introducing $M^{v}\left(s\right)$ to (45), we can write

(47) $$\begin{array}{cc} {\tilde{T}}_{n}^{v}\left(s\right) & ={\tilde{T}}_{1}^{v}\left(s\right)\left(R_{n-1}^{v}\left(s\right)-\sum_{i=1}^{n}R_{n-i}^{v}\left(s\right)G_{i}\left(s\right)\right)-\sum_{i=1}^{n}R_{n-i}^{v}\left(s\right)H_{i}\left(s\right). \end{array}$$

Now, ${\tilde{T}}_{1}^{v}\left(s\right)$ can be obtained taking (47) for n=N−1 and comparing it to ${\tilde{T}}_{N-1}^{v}\left(s\right)$ from (40) (after introducing the solutions ${\tilde{T}}_{k}^{v}\left(s\right)$ for k<N−1). Finally, we obtain the explicit solution for $T_{1}^{v}\left(s\right)$:

(48) $$\begin{array}{c} T_{1}^{v}\left(s\right)=\frac{V(s)}{W(s)}, \end{array}$$

where

(49) $$\begin{array}{cc} V(s) & =\frac{A_{0}\left(s\right)}{s}+H_{N-1}\left(s\right)-\sum_{i=1}^{N-2}A_{i}\left(s\right)\sum_{j=1}^{N-i}R_{N-i-j}^{v}\left(s\right)H_{j}\left(s\right)+\sum_{i=1}^{N-1}R_{N-i-1}^{v}\left(s\right)H_{i}\left(s\right), \end{array}$$

(50) $$\begin{array}{cc} W(s) & =\sum_{i=1}^{N-2}\left(A_{i}\left(s\right)\sum_{j=1}^{N-i}R_{N-i-j}^{v}\left(s\right)G_{j}\left(s\right)-R_{N-i-1}^{v}\left(s\right)\left(G_{i}\left(s\right)+A_{i}\left(s\right)\right)\right)+R_{N-2}^{v}\left(s\right)-G_{N-1}\left(s\right). \end{array}$$

Utilizing (48) in (29), (32) and (47), the solution for the Laplace transforms ${\tilde{T}}_{i}\left(s\right)$ and ${\tilde{T}}_{i}^{v}\left(s\right)$ for i=0,…,N−1 can be expressed as a function of the input parameters.

## 5. Numerical Examples

In this section, numerical examples are presented, and the impact of the model parameters is investigated. The CDFs $T_{k}\left(t\right)$ and $T_{k}^{v}\left(t\right)$ were computed using the Gaver–Wynn rho method of numerical Laplace transform inversion (see [31]).

Let us consider a GI/M/1/N model with single working vacations with N=30. We introduce the following notation for random variables $X_{i}$ (interarrival times in the considered model):

$$\begin{array}{cc} X_{1} & ∼Exponential(15),\\ X_{2} & ∼Uniform(0,\;2/15),\\ X_{3} & ∼Pareto(0.05,\;4),\\ X_{4} & ∼Gamma(1/3,\;1/5),\\ X_{5} & ∼Weibull(2,\;0.0752), \end{array}$$

and with $F_{i}\left(t\right)$, we denote the CDF of the corresponding variable $X_{i}.$ For all of these distributions, we have $E\left(X_{i}\right)\approx 1/15$, and therefore, in all cases, the arrival intensity is λ≈15. These distributions are used through the examples.

The parameter λ can be interpreted as the number of packets arriving to the node per second. If a single packet has size 100 B, then λ=1500B/s. Similarly, the service intensity can be converted. This way, the mean time spent in WV mode 1/α is expressed in seconds.

**Example** **1.** *The interarrival times are independent, exponentially distributed random variables. The service speed is μ=19 in normal and $\mu_{v}=9$ in WV mode.*

**Figure 1:**
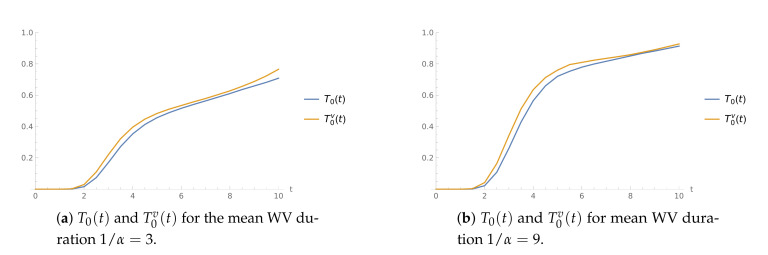
The time to buffer overflow CDFs for μ=19 and $\mu_{v}=9$.

Figure 1 shows the pairs of CDFs $T_{0}\left(t\right)$ and $T_{0}^{v}\left(t\right)$ for the mean WV period duration 1/α=3 (Figure 1a) and 1/α=9 (Figure 1b). As we could expect, the $T_{0}^{v}\left(t\right)$ values tend to be higher than those of $T_{0}\left(t\right).$ In addition, we can observe that the growth is faster in the case of longer WV.

**Example** **2.** *The interarrival times are uniformly distributed. The service speed in normal mode is μ=19.*

**Figure 2:**
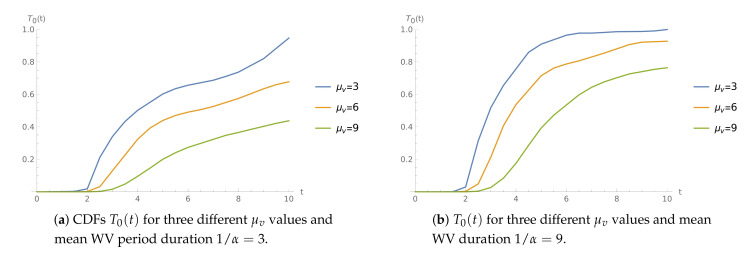
The time to buffer overflow CDFs for μ=19.

Figure 2 shows the CDFs $T_{0}\left(t\right)$ for three different values of WV service rate in case of shorter (1/α=3, Figure 2a) and longer (1/α=9, Figure 2b) WV periods. As expected, the values of $T_{0}\left(t\right)$ are greater for lower intensities $\mu_{v}.$ In addition, we can observe that they grow as the expected WV duration increases.

**Example** **3.** *The interarrival times follow a Weibull distribution. The service rates are μ=21 and $\mu_{v}=9$ in normal and WV mode, respectively.*

**Figure 3:**
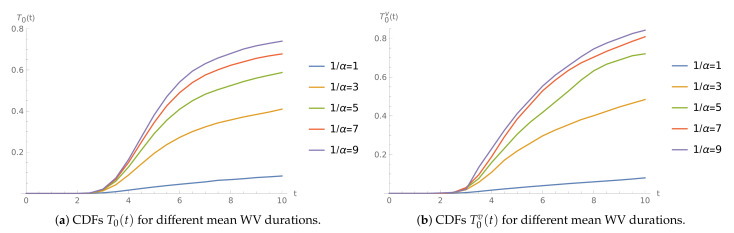
The time to buffer overflow CDFs for μ=21.

The visualization of the impact of mean WV duration is shown in Figure 3. Clearly, the WV duration parameter strongly affects the length of the period with no packet losses.

**Example** **4.** *Interarrival times follow a Pareto distribution. The normal service speed is μ=17 and is reduced to $\mu_{v}=9$ during WV.*

**Figure 4:**
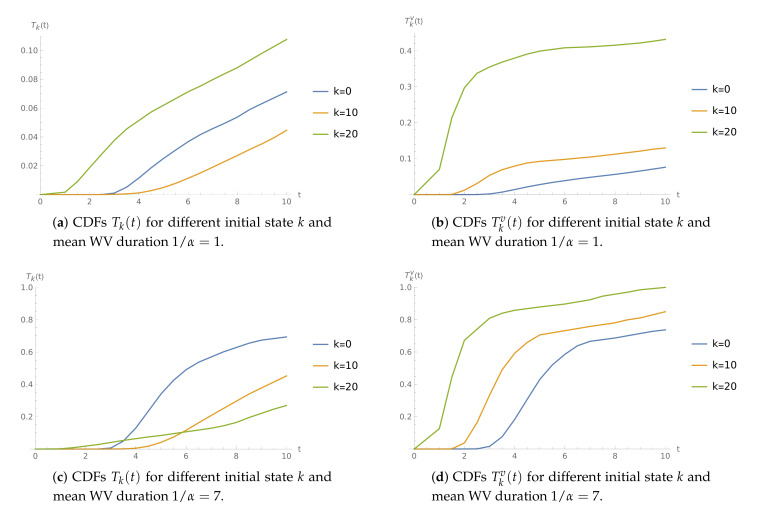
The time to buffer overflow CDFs for μ=17 and $\mu_{v}=9$.

In Figure 4, we can see that, for the longer WV periods, if the server starts in normal mode, the CDF $T_{0}\left(t\right)$ grows faster than $T_{10}\left(t\right)$ and $T_{20}\left(t\right)$ (Figure 4c). Of course, if the buffer is initially empty, the server may enter the WV period sooner, changing the workload from ρ≈0.88 to $\rho_{v}\approx 1.67.$ When the server is initially in WV mode (Figure 4b,d), the results seem more natural, i.e., the more packets that are initially present, the more probable that buffer overflow occurs before some time epoch *t*.

**Example** **5.** *The arrival intensity is λ≈15. The incoming packets are processed with rate μ=21 in normal and $\mu_{v}=12$ in WV mode.*

**Figure 5:**
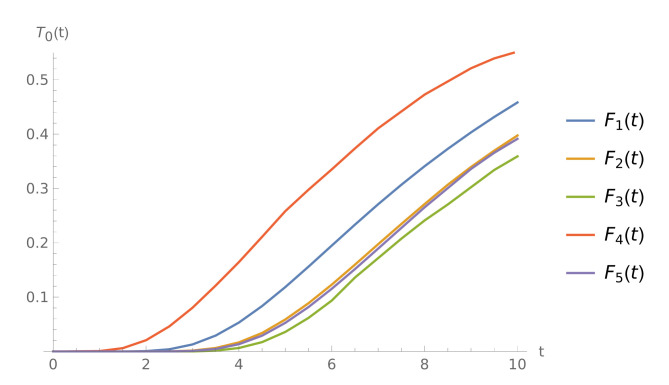
The CDFs $T_{0}\left(t\right)$ for μ=21, $\mu_{v}=12$, 1/α=9, and different types of input stream.

Figure 5 shows the CDFs $T_{0}\left(t\right)$ for different interarrival time distributions. For the gamma distributed interarrival times, the buffer tends to overflow sooner, compared to the other distributions taken into consideration. On the other side, the probability of overflow before *t* is the lowest, when the interarrival times follow a Pareto distribution. Note that $X_{4}$ has the highest variance and $X_{3}$ the lowest.

**Example** **6.** *The arrival intensity is λ≈15. The server works with intensity $\mu_{v}=9$ in WV mode, and the mean time spent in WV is 1/α=9.*

**Figure 6:**
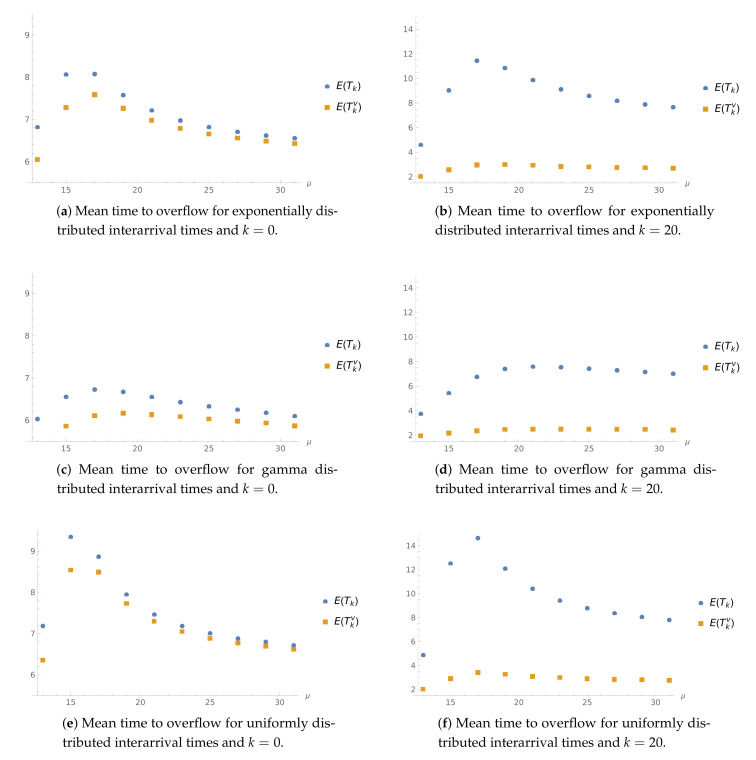
Mean value of the time to buffer overflow for different interarrival distributions with λ≈15, $\mu_{v}=9$, and 1/α=9.

In this example, we investigate the impact of μ on the expected value of the time to the first buffer overflow. As we can see in Figure 6, the expected time to the buffer overflow grows with μ for the lower range of μ, and then starts to decrease. This behaviour is linked to the fact that, as μ grows, the WV period occurrences tend to be more frequent. Another interesting observation is that, when the server starts in normal mode with 20 jobs, the mean time to the buffer overflow is greater than when it is initially empty (except for the case of μ<λ). Note that if the server starts to empty, the normal working period will end sooner; therefore, this behaviour is not surprising.

For k=20, we can observe that μ has a tenuous impact on the analysed characteristics for the server initializing its operation in WV mode. Presumably, the server would not complete the WV period before buffer overflow. Comparing the results for different interarrival distributions, namely, exponential (Figure 6a,b), gamma (Figure 6c,d), and uniform distribution (Figure 6e,f), we can draw an analogous conclusion as in the former example. For gamma distribution, the plotted expected values tend to be the lowest in the whole μ range considered.

**Example** **7.** *The arrival intensity is λ≈15. The jobs are processed with rate μ=17 in normal and $\mu_{v}=9$ in WV mode. The mean WV duration is 1/α=7.*

**Figure 7:**
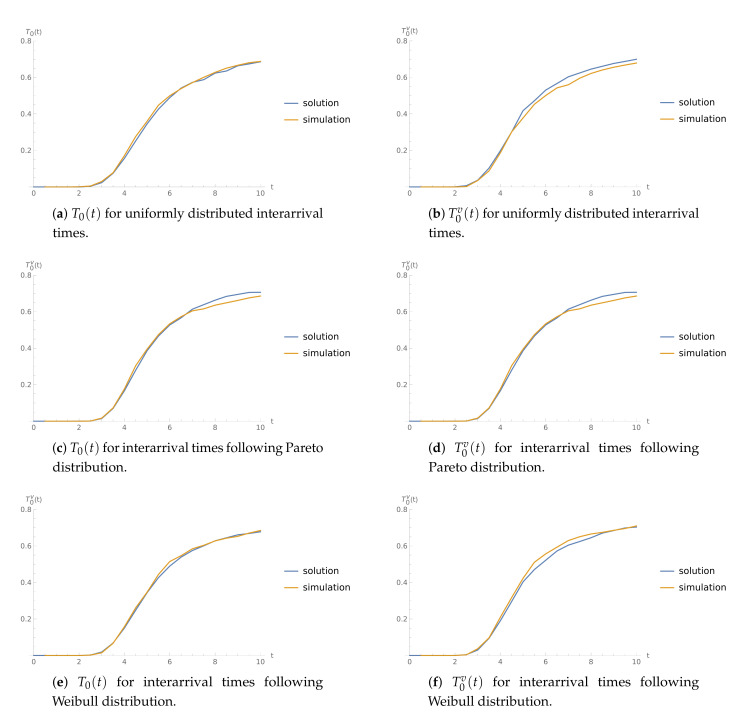
CDFs of the time to buffer overflow for $\mu =17,\;\mu_{v}=9$, and 1/α=7. Method results and simulated values.

The goal of this analysis is to validate the numerical results. Figure 7 contains the plots of CDFs $T_{0}\left(t\right)$ and $T_{0}^{v}\left(t\right)$ juxtaposed with corresponding simulated values in the case of interarrival times following uniform (Figure 7a,b), Pareto (Figure 7c,d), and Weibull distribution (Figure 7e,f). As one can note, the simulation results fit in with numerical results obtained by adopting the method described in this paper, which validate the correctness of the obtained formulae.

## 6. Conclusions

We investigated a finite-capacity queueing model with an independent general input flow, exponential service times, and a single working vacation policy. Applying an analytic approach based on the idea of an embedded Markov chain and a continuous version of the total probability law and linear algebra, the closed form representations for Laplace transforms of the time to the first buffer overflow were found for the system starting operation in both normal and working vacation mode. A detailed numerical study was conducted in which the impact of the key system parameters was analysed, such as the type of probability distribution of the interarrival times, service speeds, and single working vacation duration on the time to buffer overflow distribution.

The considered queueing system has potential applications in the modelling of energy saving modes in wireless network nodes. Energy savings can be obtained by temporarily reducing the service speed. This approach can help to reduce the latency and packet loss ratio compared to the simple vacation policy and N-policy models. The influence of the model parameters on energy usage is a subject for future research.
